# Activated Polyhydroxyalkanoate Meshes Prevent Bacterial Adhesion and Biofilm Development in Regenerative Medicine Applications

**DOI:** 10.3389/fbioe.2020.00442

**Published:** 2020-05-15

**Authors:** Sheila Piarali, Lennart Marlinghaus, Richard Viebahn, Helen Lewis, Maxim G. Ryadnov, Jürgen Groll, Jochen Salber, Ipsita Roy

**Affiliations:** ^1^Department of Surgery, Universitätsklinikum Knappschaftskrankenhaus Bochum, Ruhr-University, Bochum, Germany; ^2^Applied Biotechnology Research Group, School of Life Sciences, College of Liberal Arts and Sciences, University of Westminster, London, United Kingdom; ^3^Department of Medical Microbiology, Ruhr-University Bochum, Bochum, Germany; ^4^National Physical Laboratory, Teddington, United Kingdom; ^5^Department for Functional Materials in Medicine and Dentistry and Bavarian Polymer Institute, University of Würzburg, Würzburg, Germany; ^6^Department of Material Science and Engineering, Faculty of Engineering, University of Sheffield, Sheffield, United Kingdom

**Keywords:** polyhydroxyalkanoates, Amhelin, Dispersin B, biofilm, electrospinning

## Abstract

Regenerative medicine has become an extremely valuable tool offering an alternative to conventional therapies for the repair and regeneration of tissues. The re-establishment of tissue and organ functions can be carried out by tissue engineering strategies or by using medical devices such as implants. However, with any material being implanted inside the human body, one of the conundrums that remains is the ease with which these materials can get contaminated by bacteria. Bacterial adhesion leads to the formation of mature, alive and complex three-dimensional biofilm structures, further infection of surrounding tissues and consequent development of complicated chronic infections. Hence, novel tissue engineering strategies delivering biofilm-targeted therapies, while at the same time allowing tissue formation are highly relevant. In this study our aim was to develop surface modified polyhydroxyalkanoate-based fiber meshes with enhanced bacterial anti-adhesive and juvenile biofilm disrupting properties for tissue regeneration purposes. Using reactive and amphiphilic star-shaped macromolecules as an additive to a polyhydroxyalkanoate spinning solution, a synthetic antimicrobial peptide, Amhelin, with strong bactericidal and anti-biofilm properties, and Dispersin B, an enzyme promoting the disruption of exopolysaccharides found in the biofilm matrix, were covalently conjugated to the fibers by addition to the solution before the spinning process. *Staphylococcus epidermidis* is one of the most problematic pathogens responsible for tissue-related infections. The initial antibacterial screening showed that Amhelin proved to be strongly bactericidal at 12 μg/ml and caused >50% reductions of biofilm formation at 6 μg/ml, while Dispersin B was found to disperse >70% of pre-formed biofilms at 3 μg/ml. Regarding the cytotoxicity of the agents toward L929 murine fibroblasts, a CC_50_ of 140 and 115 μg/ml was measured for Amhelin and Dispersin B, respectively. Optimization of the electrospinning process resulted in aligned fibers. Surface activated fibers with Amhelin and Dispersin B resulted in 83% reduction of adhered bacteria on the surface of the fibers. Additionally, the materials developed were found to be cytocompatible toward L929 murine fibroblasts. The strategy reported in this preliminary study suggests an alternative approach to prevent bacterial adhesion and, in turn biofilm formation, in materials used in regenerative medicine applications such as tissue engineering.

## Introduction

Within regenerative medicine applications, the failure of implantable *in situ* tissue engineering scaffolds, that should replace missing or damaged tissue and recruit cells from the surrounding tissues of the host, bears generally the probability of bacterial adhesion to its external and internal surface and subsequent biofilm formation at the time point of implantation or even later by hematogenous spread. Finally, this leads to a chronically colonized biomaterial scaffold with the intrinsic capability to induce an infection at any later time (Brancatisano et al., 2014; Segev-Zarko et al., 2015; Batoni et al., 2016).

Biofilms can tolerate various types of physicochemical treatments including UV light, heavy metals, acidity, changes in hydration or salinity, and phagocytosis. Additionally, biofilms typically resist antibiotics even at high concentrations (Lebeaux et al., 2014). The complexity of the biofilm structure, decreased growth rates, secretion of enzymes capable of degrading antibiotics, horizontal gene transfer and communication via quorum sensing systems are just few of the main factors involved in the resistance of biofilms against antibiotics (Donlan and Costerton, 2002; Wi and Patel, 2018). Clearly this leads to many therapeutic difficulties and most of the times the solution involves surgical removal of all foreign material (e.g., medical device, implant or scaffold) and comprehensive debridement of the infected tissue (Zimmerli and Moser, 2012). This, along with the lack of treatment options against biofilms, emphasizes the need to develop new locally acting antibacterial, anti-adhesive and anti-biofilm materials in the regenerative medicine field.

Tissue formation can be prompted by using polymeric scaffolds with specific characteristics. For instance, fibrous polymeric scaffolds are extremely attractive for tissue engineering applications due to their topographical similarities with the fibrillae of the extracellular matrix (ECM) and can be produced using a simple, cost-effective, and versatile approach such as electrospinning (Repanas et al., 2016).

In terms of the choice of the polymeric material, there are a variety of natural and synthetic polymers suitable for the development of tissue engineering scaffolds, development of medical implants and antimicrobial surfaces. Polyhydroxyalkanoates (PHAs) are highly biocompatible and naturally occurring bacteria-derived polyesters and are produced by bacterial fermentation (Lebeaux et al., 2014; Li et al., 2016). A variety of PHAs with different properties and degradation rates can be obtained by changing the conditions of the fermentation process. Furthermore, PHAs can be processed using several techniques such as solvent casting, dip molding, 3D printing and electrospinning, making them attractive candidates for a variety of medical applications (Reis et al., 2008; Roy and Visakh, 2014).

Regarding the fiber activation against bacteria, it can be done by covalent attachment of antibacterial agents such as enzymes and antimicrobial peptides.

Dispersin B (DB) is a 42 kDa glycoside hydrolase produced by a Gram-negative bacteria *Aggregatibacter actinomycetemcomitans*, a human periodontal pathogen. DB is capable of degrading polysaccharides present in the biofilm matrix of many bacterial strains, resulting in the detachment of the bacterial cells (swarming phenotypes) and their release into the environment. More specifically, it degrades cationic extracellular polysaccharides, namely β-1,6-poly-N-acetylglucosamine (PNAG), that work as adhesins and mediate the attachment of bacteria to abiotic surfaces (Kaplan, 2009, 2010).

Antimicrobial peptides (AMPs), on the other hand, have been extensively explored as an alternative to conventional antibiotics due to their exceptionally broad spectrum of activity against bacteria, bactericidal efficacy at low concentrations as well as their multiple modes of action (Onaizi and Leong, 2011). AMPs vary by size, comprising 10–50 amino-acid residues, net charge, primary and secondary structures. However, these peptides also share common characteristics. Most of them are cationic and fold upon binding to anionic microbial membranes into amphipathic structures by clustering cationic and hydrophobic residues in two distinct faces (Ebbensgaard et al., 2015). Because their action is folding mediated, it can be controlled in a dose dependent and reversible manner (Ryan et al., 2013). Once in the membranes, the peptides arrange into membrane-disrupting pores or channels that destabilize microbial cells, resulting in cell lysis and death (Powers and Hancock, 2003; Brogden, 2005; Hancock and Sahl, 2006). One of the reasons for conventional antibiotics to fail against biofilms is their inability to target cells with low metabolic activity. Membrane integrity is essential to bacteria regardless of their metabolic state, whilst AMPs do not differentiate between dormant, dividing or mature bacterial cells, killing all. Therefore, the use of AMPs against cells that are resistant to antibiotics presents an effective strategy to prevent biofilm formation or treat established biofilms. However, despite their activity, naturally occurring AMPs pose some limitations regarding their toxicity against eukaryotic cells and therefore synthetic AMPs are now gaining more attention as they can show reduced cytotoxicity (Steinstraesser et al., 2002). Antimicrobial helix insert (here termed as Amhelin) is a novel *de novo* sequence designed as an archetypal antimicrobial peptide. The peptide folds into an amphipathic α helix in microbial lipid bilayers where it forms progressively expanding pores (Rakowska et al., 2013). Unlike many AMPs, Amhelin does not cause hemolytic effects either, with median lethal concentrations (LC_50_) against human erythrocytes significantly exceeding the measured CC_50_ (Ryadnov et al., 2009; Li et al., 2017).

Antimicrobial peptides or enzymes are normally attached to fiber surfaces post-processing by covalent attachment, which usually involves several activation steps and includes the use of chemical cross-linkers. An alternative approach was reported by Grafahrend et al., 2011, where they used an amphiphilic six-armed star-shaped poly(ethylene oxide-stat-propylene oxide) containing reactive isocyanate groups at the distal ends of the polymer chains, NCO-sP(EO-stat-PO), as an additive to the PLGA spinning solution. The presence of the isocyanate groups facilitated the covalent coupling of cell adhesion peptides by addition of these peptides to the spinning solution, thus resulting in the production of surface-activated fibers in a single step (Grafahrend et al., 2011). This approach has recently been used to produce artificial basement-membranes as a component for the generation of *in vitro* skin tissue and expanded for multi-functionalization with peptides and antibodies for immunomodulatory properties (Wistlich et al., 2015; Rossi et al., 2016).

In this study, we transferred this strategy to PHAs and developed PHA-[NCO-sP(EO-stat-PO)] based blend fibers which we functionalized with membrane targeting Amhelin and with the biofilm-disrupting enzyme DB. These substrates allowed us to study the effect of fiber functionalization against bacterial adhesion and the establishment of predominantly polysaccharide-based biofilms, such as staphylococcal biofilms, whilst still allowing for eukaryotic cell attachment. Our experiments demonstrate that the resulting material combines anti-adhesive and anti-biofilm properties, and hence holds huge promise for medical applications.

## Materials and Methods

### Materials

Chemicals, reagents and materials were obtained from Sigma-Aldrich Company Ltd., (United Kingdom and Germany), Thermo Fisher Scientific (United Kingdom and Germany), VWR International (United Kingdom), and Carl Roth GmbH + Co., KG (Germany).

### Polymers and Antibacterial Agents

Polyhydroxyalkanoates were produced by bacterial fermentation under nitrogen limiting conditions and with glucose as the carbon source using *Pseudomonas mendocina* CH50 at the Department of Life Sciences in the University of Westminster, London, United Kingdom.

Amphiphilic six-armed star-shaped poly(ethylene oxide-stat-propylene oxide) containing reactive isocyanate groups at the distal ends of the polymer chains, NCO-sP(EO-stat-PO), was provided by Prof. Jürgen Groll, Department for Functional Materials in Medicine and Dentistry and Bavarian Polymer Institute, University of Würzburg, Würzburg, Germany.

Dispersin B was provided by Kane Biotech Inc., (Canada) in vials containing a buffer solution consisting of 50 mM phosphate buffer (pH 5.8) with 100 mM sodium chloride and 50% glycerol at a concentration of 475 μg/mL per vial. Amhelin was synthesized by the National Physical Laboratory (United Kingdom) and was provided in the form of a lyophilized powder. The peptide was kept in the freezer, at −20°C for routine usage.

### Bacterial Strains

All antibacterial tests were conducted using the biofilm forming strain *Staphylococcus epidermidis* (ATCC^®^ 35984^TM^), purchased from the American Type Culture Collection (ATCC). The bacterial culture was stored as glycerol stocks in the freezer, at −20°C, for routine usage or at −80°C for longer storage duration.

The preparation of the inoculum for all antibacterial tests was done by culturing bacteria overnight on blood agar plates at 37°C for 16 h. Using a sterile loop, a single colony was re-suspended in a glass tube containing 2 mL of 0.9% NaCl and using a densitometer, the bacterial suspension was adjusted to McFarland 0.5 (10^8^ CFU/mL). According to each experiment, the bacterial suspension was further diluted in Mueller-Hinton-Broth (MHB) to the desired concentration.

### Cell Lines and Cell Culture Materials

L929 murine fibroblasts were obtained from DSMZ (German Collection of Microorganisms and Cell Cultures GmbH). Roswell Park Memorial Institute (RPMI) medium, L-glutamine, 10% fetal bovine serum and 1% Penicillin-Streptomycin (10.000 U/mL Penicillin, 10 mg/mL Streptomycin) were obtained from PAN Biotech (Germany), as well as trypsin 0.25%/1 mM EDTA and Dulbecco’s Phosphate-Buffered Saline (DPBS) without calcium and magnesium. Triton^®^ X-100 lysis solution and CellTiter-Blue^®^ Cell Viability Assay Kit were purchased from Promega. Live/dead Cell Staining Kit II was purchased from PromoKine.

L929 murine fibroblasts were cultivated in complete Roswell Park Memorial Institute medium, containing L-glutamine, 10% fetal bovine serum and 1% Penicillin-Streptomycin (10.000 U/mL Penicillin, 10 mg/mL Streptomycin) (cRPMI) and maintained in a humidified atmosphere at 5% CO_2_ in air at 37°C. When cells reached 70–80% confluency, they were detached using a trypsin/EDTA solution, collected, and the number of cells was adjusted in a cell suspension according to each experiment. In order to avoid variations in cell viability tests, cells were used until passage 10.

### Minimum Inhibitory and Minimum Bactericidal Concentrations of Amhelin Against Bacteria

The minimum inhibitory concentration (MIC) and the minimum bactericidal concentration (MBC) of Amhelin was determined against *S. epidermidis* (ATCC^®^ 35984^TM^) according to the ISO 20776-1:International Organization for Standardization [ISO], 2006 guidelines in 96 tissue-culture polystyrene (TCPS) microplates (ISO 20776-1:International Organization for Standardization [ISO], 2006(E)).

Briefly, Amhelin was prepared by making a stock solution of 1 mg/mL in sterile Milli Q water. The stock solution was further diluted in MHB to a final concentration of 200 μg/mL. A fresh bacterial suspension of 5 × 10^5^ CFU/mL was prepared in MHB and serial 2-fold dilutions were prepared by incubating 50 μL of the bacterial suspension with 50 μL of the Amhelin solution. The plates were incubated at 37°C for 24 h and the MIC was determined as the lowest concentration of Amhelin that completely inhibited bacterial growth by observing the turbidity of the wells by naked eye. Wells containing 100 μL of MHB without bacteria were considered as negative controls and wells containing 100 μL of MHB with bacteria were considered as the growth controls. The experiments were performed in three independent experiments, in triplicates (*N* = 3, *n* = 3). The MBC was determined directly after identifying the MIC. For that, the supernatant of the wells corresponding to the MIC and of three concentrations above were removed and spread homogeneously onto blood agar plates with the help of a sterile loop. The plates were incubated at 37°C for 24 h. After the incubation period, the number of colonies were counted and compared to the number of colonies obtained in the growth controls at the beginning of the experiment. The MBC was determined as the lowest concentration of the compound that killed 99.9% or more of the bacteria, relative to that in the initial inoculum. The experiments were performed in three independent experiments, in duplicates (*N* = 3, *n* = 2).

### Inhibition of Biofilm Formation by Amhelin

The ability of Amhelin to inhibit biofilm formation was evaluated by quantifying the amount of biofilm formed after a 24-hour incubation of bacteria with increasing concentrations of Amhelin, by adapting the protocol described by Kiran et al. (2008). Similar to the previous method, serial dilutions of Amhelin were incubated with a fresh bacterial suspension of 5 × 10^5^ CFU/mL. After a 24-hour incubation period, the supernatant was entirely removed from the wells and the wells were carefully washed twice with 100 μL of DPBS and dried at room temperature. To fix the biofilms, 100 μL of 100% ethanol were added to each well and the plate was incubated for 15 minutes at room temperature. The ethanol was removed, and the biofilms were stained with 100 μL of a 0.1% safranin-O solution and incubated for 5 min at room temperature followed by a washing step with 100 μL of DPBS. Finally, the stained biofilms were re-suspended in 100 μL of 1% sodium dodecyl sulfate (SDS) and the amount of biofilm was quantified by measuring the optical density of the dissolved safranin-O at 495 nm (OD_495_). The experiments were performed in three independent experiments, in triplicates (*N* = 3, *n* = 3).

### Dispersion of Pre-established Biofilms by Dispersin B

The ability of DB to disperse 24-hour pre-formed biofilms was tested against *S. epidermidis* (ATCC^®^ 35984^TM^) in a 96 well TCPS microplate according to Marcano et al. (2015).

Each well was loaded with 100 μL of a bacterial suspension of 5 × 10^5^ CFU/mL and incubated at 37°C for 24 h. After this period, the supernatant was carefully removed and filled with 100 μL of serially diluted concentrations of DB in MHB and incubated at 37°C for 24 h. After the incubation period, the supernatant was removed, and the quantification of the biofilm biomass was performed as described in section “Inhibition of Biofilm Formation by Amhelin.” The experiments were performed in three independent experiments, in triplicates (*N* = 3, *n* = 3).

### *In vitro* Direct Cytotoxicity Evaluation of Amhelin and Dispersin B

The cytotoxicity of Amhelin and DB were evaluated by performing a direct cytotoxicity study and by calculating the cell viability using the CellTiter-Blue^®^ Cell Viability Assay. The experiment was performed following the ISO 10993-5 guidelines [ISO 10993- 5:International Organization for Standardization [ISO], 2009(E)] in 96 well TCPS microplates.

The CellTiter-Blue^®^ Cell Viability Assay was used to estimate the number of viable cells after their exposure to increasing concentrations of Amhelin and DB for a period of 24 h. The assay is based on the capability of metabolically active cells, that is, living cells to convert the redox dye resazurin into a fluorescent resorufin which is analyzed using a plate-reading fluorometer. Briefly, on a 96 well TCPS microplate, 100 μL of a cell suspension containing 10^4^ cells was seeded per well and incubated in a humidified atmosphere at 5% CO_2_ in air, at 37°C, for 24 h, to promote cell attachment. On the next day, the cell media was discarded from each well and 100 μL of increasing concentrations of each agent, in cRPMI, were added to each well. The cells were incubated in a humidified atmosphere at 5% CO_2_, in air, at 37°C, for another 24 h. TCPS was used as negative control and the positive control was composed of lysed cells after being treated with 2 μL of 9% Triton^®^ X-100 (Promega) for 5 min after the 24-hour incubation period. After the incubation period, the cell supernatant was removed from the wells and 120 μL of the assay reagent (CellTiter-Blue diluted with cRMPI) was added to each well and incubated in a humidified atmosphere at 5% CO_2_ in air at 37°C, for 2 h. After the incubation period, the supernatant was transferred to a black 96 well TCPS microplate and the fluorescence was read at 560 nm. The experiments were performed in two independent experiments, in quadruplicate (*N* = 2, *n* = 4).

### Fabrication of Aligned and Bead-Less PHA Fibers by Electrospinning

The production of PHA fibers was carried out using a custom-built electrospinning setting composed of a high voltage power supply, a syringe pump and a rotating steel cylindrical collector. A 2 mL polypropylene syringe (BD Discardit^TM^ II) equipped with a 23-gauge steel needle with a blunt tip was used to fabricate the fibers. The polymer solutions were fed free of air bubbles to the 2 mL syringe and the fibers were electrospun at a flow rate of 5 mL/h, with a 14 kV voltage, a needle-to-collector distance of 14 cm and a rotator speed of 1000 rpm. All fibers were electrospun to 1 cm diameter glass coverslips. After the electrospinning process, a drying period of at least 24 h was used to allow the complete evaporation of any remaining solvent from the fibers inside a biological safety cabinet, in ambient conditions. Subsequently, the fibers were sterilized under the UV light – UVGI at short-wavelength ultraviolet light (UV-C, mercury-based lamp emitting UV light of 253.7 nm) for 30 min. PHAs were dissolved in a 60:40 chloroform/acetone solution at 20 w/v% and the fibers were electrospun directly from this solution.

The surface morphology of the fibers was analyzed by scanning electron microscopy (SEM). The images were taken with an acceleration voltage of 5 kV at a 10 mm working distance. The analysis was carried out at the Analytical Chemistry – Center for Electrochemical Sciences (CES) at the Ruhr-University Bochum, Germany.

### Surface Activation of PHA Fibers

The activation of the PHA fibers was carried out using NCO-sP(EO-stat-PO), in order to promote the covalent coupling of active agents. For that, PHA was dissolved in a 60:40 chloroform/acetone solution at 20 w/v% and NCO-sP(EO-stat-PO) was dissolved in acetone at 5 w/v%. Once each solution was homogeneous, both solutions were mixed and vortexed vigorously. PHA-NCO-sP(EO-stat-PO) fibers were electrospun directly from this solution.

Bioactivated fibers were produced by coupling Amhelin and DB to PHA-NCO-sP(EOstat-PO) fibers. Briefly, using a ratio of 1:10, Amhelin:NCO-sP(EO-stat-PO), the required amount of peptide in the form of lyophilized powder was dissolved in a solution containing 50 μg/mL of DB. The solution was mixed and vortexed vigorously and fibers were electrospun directly from this solution.

Using the same conditions described in section “Fabrication of Aligned and Bead-Less PHA Fibers by Electrospinning,” fibers were electrospun onto 1 cm diameter glass coverslips. After the electrospinning process, a drying period of at least 24 h was used to allow the complete evaporation of any remaining solvent from the fibers, inside a biological safety cabinet, in ambient conditions. Subsequently, the fibers were sterilized under the UV light - UVGI at short-wavelength ultraviolet (UV-C, mercury-based lamp emitting UV light at the 253.7 nm line) for 30 min.

The set of fibers produced in this study were PHA fibers, PHA-NCO-sP(EO-stat-PO) fibers and PHA-NCO-sP(EO-stat-PO) + Amhelin + DB fibers.

### Water Contact Angle of Activated Fibers

The wettability of the modified fibers was evaluated by the static sessile drop method using a custom-built contact angle goniometer. On an average, 30 μL of deionized water was dropped onto the surface of the fibers with the help of a syringe. The contact angle was measured using a custom-built software. Analysis was carried out by the Analytical Chemistry – Biointerfaces group located at the Ruhr-University Bochum, Germany. The measurements were performed in triplicates (*n* = 3).

Further experiments were carried out with PHA and PHA-NCO-sP(EO-stat-PO) fibers on glass coverslips using different pre-conditioning procedures to verify the WCAs measured on the dried fiber mesh surfaces. The substrates were incubated in a deionized water-saturated atmosphere at 37°C for 4 h. Afterward, WCA measurements were carried out as previously described but this time as two independent experiments, in 10-fold (*N* = 2, *n* = 10).

### Anti-adhesive Properties of Activated Fibers

To evaluate the anti-adhesion properties of the modified fibers, glass coverslips containing the fibers were placed in a sterile 24-well polystyrene microtiter plate and each well was inoculated with 1 ml of a bacterial suspension containing 5 × 10^5^ CFU/mL. The plate was incubated at 37°C for 24 h in under static conditions. After the incubation period, the liquid content from the wells was removed and each fiber coated glass coverslip was washed twice with PBS to remove loosely adhered bacterial cells. Each sample was placed in sterile tubes containing 10 mL of 0.9% sodium chloride and sonicated in a sonication bath for 5 min followed by vortex mixing for 10 s to detach the bacterial cells. The procedure was repeated three times. The sonicates were serially diluted in 0.9% sodium chloride and 100 μL of each dilution were plated onto blood agar plates. After an 18-hour incubation at 37°C, the colonies were counted and expressed as CFU/ml. To complement the test, after the sonication procedure, the glass coverslips were immersed in an 100% ethanol solution for 15 min, then stained with a 0.1% safranin-O solution and incubated for 5 min at room temperature, followed by two-washing steps with DPBS. The stained-glass coverslips were dried overnight and imaged using a Panasonic DMC-FZ100. The experiments were performed in three independent experiments, in triplicates (*N* = 3, *n* = 3).

### *In vitro* Direct Cytotoxicity Evaluation of Activated Fibers

To evaluate the fiber cytocompatibility, the CellTiter-Blue^®^ Cell Viability Assay was performed. Briefly, sterile glass coverslips containing the modified fibers were placed in a 24-well polystyrene microtiter plate. A L929 cell suspension containing 5 × 10^4^ cells was seeded per well on top of each glass coverslip and incubated in a humidified atmosphere of 5% CO_2_ in air at 37°C for 24 h. Following the incubation period, the cell supernatant was removed and 250 μL of assay reagent (CellTiter-Blue diluted with cRMPI) was added to each well and incubated in a humidified atmosphere of 5% CO_2_ in air at 37°C, for 2 h. After the incubation period, 120 μL of each well were transferred to a black 96-well polystyrene microtiter plate and the fluorescence was read at 560 nm. Tissue Culture Plastic (TCP) and non-modified fibers were used as the positive controls and the negative control was a lysis solution composed of Triton^®^ X-100 used to cause cell lysis.

### Live-Dead Staining of L929 Murine Fibroblasts on Activated Fibers

In order to correlate the data obtained via the CellTiter-Blue^®^ Cell Viability Assay, a fluorescent cell staining was performed using a two-color fluorescent staining. Calcein-AM was used to stain live cells in green and Ethidium homodimer-III (EthD-III) was used to stain dead cells in red. Living cells are characterized by the presence of intracellular esterase activity which can convert non-fluorescent Calcein-AM to green fluorescent Calcein. On the other hand, EthD-III can enter the cells that have a damaged membrane and upon binding to nucleic acids can produce red fluorescence. After growing the cells on top of the modified fibers, the culture medium was removed, and each well was washed with DPBS. A staining solution of 2 μM Calcein-AM/4 μM EthD-III was prepared in PBS and 480 μL of the staining solution were added directly to each well. The cells were imaged using an inverted fluorescence microscope (Olympus IX51).

### Statistical Analysis

Experiments were performed on freshly prepared samples and the results were reported as averages and standard deviations of these measurements. The statistical analysis was done using GraphPad Prism 5 version 5.00. Data were compared using unpaired *t*-test or a one-way analysis of variance (ANOVA) Tukey and Dunnett’s test. Differences were considered significant for *p*-values lower than 0.05 (*p* < 0.05).

## Results

### Antibacterial Characterization of Amhelin Against *Staphylococcus epidermidis* (ATCC^®^ 35984^TM^)

As a starting point, the antibacterial properties of Amhelin were tested against *S. epidermidis* (ATCC^®^ 35984^TM^) by determining the MIC and MBC, respectively. The bacterial strain was cultured in the presence of increasing concentrations of Amhelin for a period of 24 h. The peptide concentration that completely inhibited visible bacterial growth was determined as the MIC, while the peptide concentration that resulted in a reduction of 99.9% of CFUs relative to that in the initial inoculum was determined as the MBC. The MIC and the MBC of Amhelin against *S. epidermidis* (ATCC^®^ 35984^TM^) were found to be 6 and 12 μg/mL, respectively (Table 1).

**TABLE 1:** MIC and MBC of Amhelin against *S. epidermidis* (ATCC^®^ 35984^TM^).

**Amhelin**	**MIC**	**MBC**
*S. epidermidis* (ATCC^®^ 35984^TM^)	6 μg/mL	12 μg/mL

**FIGURE 1:**
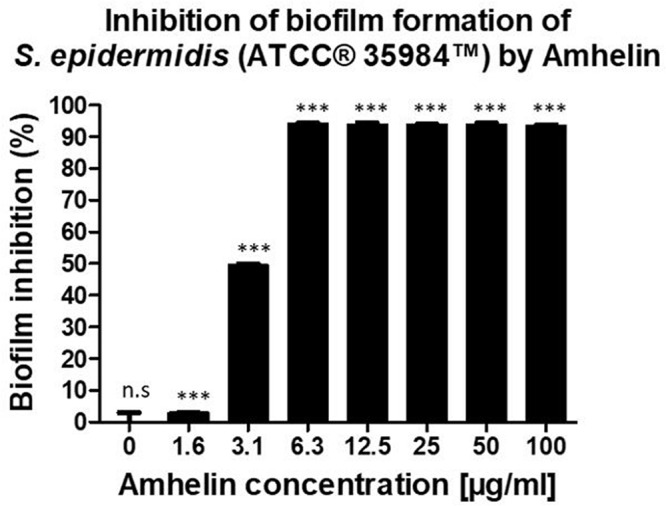
Percentage of inhibition of biofilm formation of *S. epidermidis* (ATCC^®^ 35984^TM^) by Amhelin when exposed to increasing concentrations of Amhelin for a period of 24 h. The biofilm growth control is represented by the column in which no Amhelin was added. The experiments were performed in three independent experiments, in triplicates (*N* = 3, *n* = 3). ^∗∗∗^*p* < 0.0001 and n.s indicates no statistical difference when compared with biofilm growth control.

Furthermore, the anti-biofilm properties of Amhelin were evaluated by quantifying the biofilm biomass that was produced after a 24-hour bacterial exposure to increasing concentrations of Amhelin. The biofilm biomass was quantified using a safranin staining method and the results were expressed as a percentage of biofilm inhibition compared to a typical biofilm growth control. With the lowest peptide concentration, that is 1 μg/mL, biofilm formation was inhibited by only 3%. A more significant effect was observed with a peptide concentration of 3 μg/mL, where 50% of biofilm formation was inhibited. With concentrations equal or higher to the MIC, that is 6 μg/mL, biofilm formation was inhibited by more than 90% (Figure 1).

### Dispersin B Is Capable of Detaching Pre-formed Biofilms

The ability of DB to disperse pre-formed biofilms was tested against *S. epidermidis* (ATCC^®^ 35984^TM^) by incubating 24-hour pre-formed biofilms with increasing concentrations of DB for a period of 24-hours and by quantifying the biofilm biomass that remained intact after the incubation period, using a safranin staining method. The results were expressed as a percentage of biofilm dispersal compared to a typical biofilm growth control.

**FIGURE 2:**
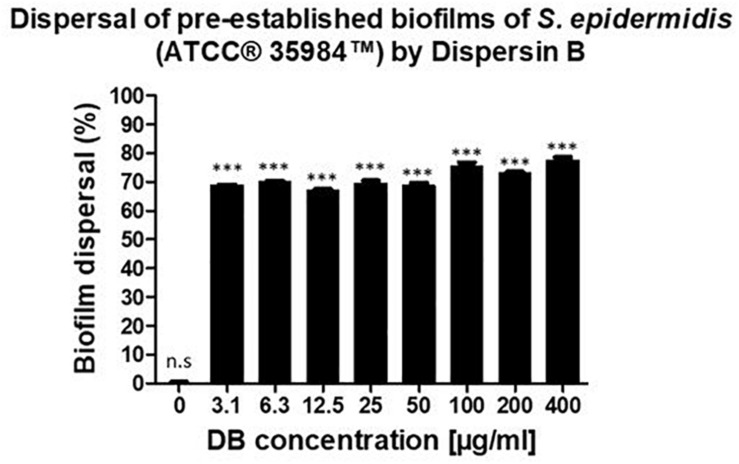
Percentage of biofilm dispersal as a result of the incubation of 24-hour pre-established biofilms of *S. epidermidis* (ATCC^®^ 35984^TM^) with increasing concentrations of DB for 24 h. The biofilm growth control is represented by the column in which no DB was added. The experiments were performed in three independent experiments, in triplicates (*N* = 3, *n* = 3). ^∗∗∗^*p* < 0.0001 and n.s indicates no statistical difference when compared with biofilm growth control.

A similar dispersal activity was observed for all the testing DB concentrations. With concentrations up until 50 μg/mL, 70% of pre-established biofilms were dispersed. By increasing the DB concentrations to 100, 200, and 400 μg/mL, pre-established biofilms were dispersed by 76, 73, and 78%, respectively (Figure 2).

### L929 Murine Fibroblasts Can Grow in the Presence of Amhelin and Dispersin B

In order to evaluate the cytotoxicity of each of the active agents, cytocompatibility studies were performed by incubating pre-adhered L929 murine fibroblasts with increasing concentrations of either Amhelin or DB for a period of 24 h and by evaluating the percentage of cell viability after the incubation period. Each graph shows the percentage of viable L929 murine fibroblasts after being exposed to increasing concentrations of Amhelin and DB for 24 h in comparison to a cell growth control.

With the results obtained, it was possible to determine the 50% cytotoxicity concentration (CC_50_), that is, the concentration resulting in a 50% viability of L929 murine fibroblasts of each agent as well as to compare the concentrations of the agents at which high antibacterial performance was obtained with the L929 cell viability. Amhelin showed a CC_50_ of 143 μg/mL while DB showed a CC_50_ of 134 μg/mL.

**FIGURE 3:**
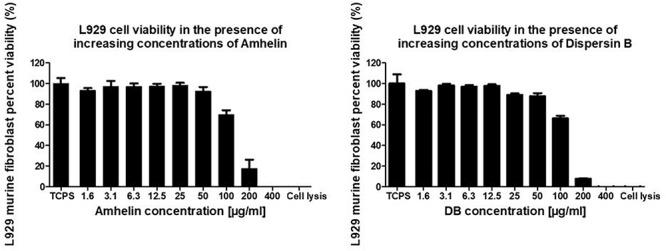
Percentage of cell viability of L929 murine fibroblasts, after growing in the presence of increasing concentrations of Amhelin and DB, for a period of 24 h. A typical cell growth control and a positive control were considered and are represented as TCPS and cell lysis, respectively.

The concentrations at which Amhelin showed strong bactericidal and anti-biofilm properties, that is, 12 and 3 μg/mL resulted in a L929 cell viability higher than 90%. In the case of DB, with concentrations up until 50 μg/mL, a cell viability higher than 70% was obtained (Figure 3).

### Fabrication of Polyhydroxyalkanoate- Based Fibers

**FIGURE 4:**
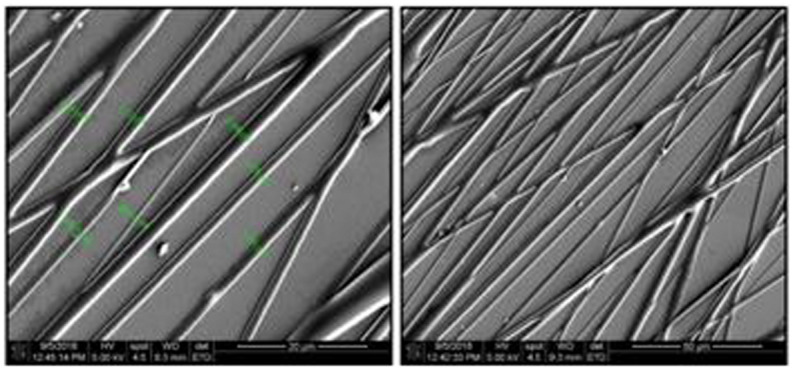
SEM micrographs of PHA fibers using a 20 w/v% polymer concentration in a 60/40, chloroform/acetone solution, 14 kV, 5 mL/h flow rate, needle-to-collector distance of 14 cm and target’s rotation speed of 1000 rpm. Magnification of micrographs on the left-side of 1500× and scale bar of 20 μm. Magnification of micrographs on the right-side of 500× and scale bar of 50 μm. Low fiber density samples were used for imaging purposes only.

The electrospinning process resulted in well aligned and bead-less fibers with diameters ranging from 500 nm to 2.5 microns (Figure 4).

### Water Contact Angle of Activated Fibers

To evaluate the wettability changes after the functionalization process, wettability studies were conducted by measuring the water contact angle of PHA-NCO-sP(EO-stat-PO) fibers, PHA-NCO-sP(EO-stat-PO) + Amhelin + DB fibers and compared to the water contact angle of PHA disks obtained by a solvent casting method and PHA fibers. PHA disks, PHA fibers, PHA-NCO-sP(EO-stat-PO) fibers were also pre-incubated in a deionized water-saturated atmosphere at 37°C for 4 h to assess the changes in wettability.

**FIGURE 5:**
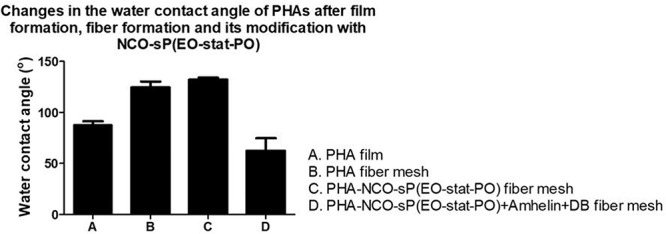
Water contact angle of PHA disks, PHA fibers, PHA-NCO-sP(EO-stat-PO) fibers and PHA-NCO-sP(EO-stat-PO) + Amhelin + DB fibers.

**FIGURE 6:**
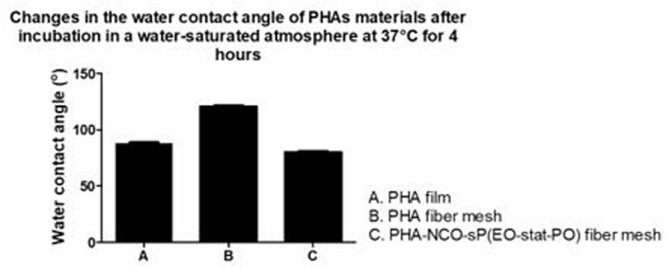
Water contact angle of PHA disks, PHA fibers, PHA-NCO-sP (EO-stat-PO) fibers after incubation in a deionized water-saturated atmosphere at 37°C for 4 h.

Polyhydroxyalkanoates disks exhibited a water contact angle of 88^*o*^ confirming the hydrophobic nature of the material. After fiber formation, PHA fibers showed a significant increase in hydrophobicity, with a water contact angle of 124°. A further increase in hydrophobicity was observed for PHA-NCO-sP(EO-stat-PO) fibers which showed a water contact angle of 132°. On the other hand, PHA-NCO-sP(EO-stat-PO) + Amhelin + DB fibers showed a water contact angle of 62° (Figure 5). The pre-incubation in a deionized water-saturated atmosphere, at 37°C, for 4 h, showed that the PHA disks and PHA fibers maintained their wettability properties, showing water contact angles of 88 and 120°, respectively. On the other hand, PHA-NCO-sP(EO-stat-PO) fibers showed a decrease in the water contact angle, to a value of 80° (Figure 6).

### *In vitro* Direct Cytotoxicity Studies on Activated Fibers

In order to evaluate the cytotoxicity of the functionalized fibers against mammalian cells, L929 murine fibroblasts were seeded on the surface of PHA fibers, and on modified PHA fibers for a period of 24 h. The cytotoxicity of each material was evaluated by calculating the percentage cell viability after the incubation period in comparison to the growth control. Additionally, a fluorescent cell staining was carried out to observe the presence of live and dead cells on the fibers.

**FIGURE 7:**
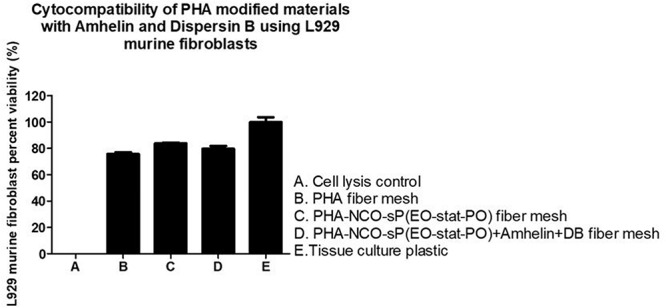
Percentage of cell viability of L929 murine fibroblasts after its seeding on the surface of PHA fibers, PHA-NCO-sP(EO-stat-PO) fibers and PHA-NCO- sP(EO-stat-PO) + Amhelin + DB fibers for a period of 24 h. A typical cell growth control and a positive control were considered and are represented by TCPS and cell lysis, respectively.

The graph shows the percentage of viable L929 murine fibroblasts that grew in the presence of the different fibers in comparison to the cell growth control, TCPS, after an incubation period of 24 h. A cell viability of 76 and 84% was observed for PHA and PHA-NCO-sP(EO-stat-PO) fibers, respectively. PHA-NCO-sP(EO-stat-PO) + Amhelin + DB fibers exhibited a cell viability of 80% (Figure 7).

**FIGURE 8:**
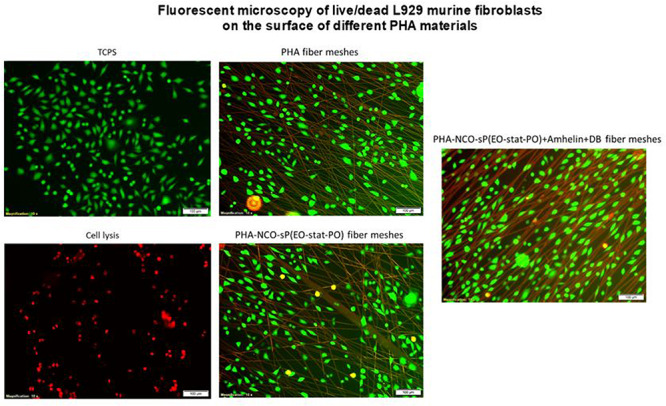
Fluorescence micrographs of L929 murine fibroblasts grown on top of PHA fibers, PHA-NCO-sP(EO-stat-PO) fibers and PHA-NCO-sP(EO-stat-PO) + Amhelin + DB fibers. The dead cells are represented in red and live cells are represented in green. Magnification of micrographs of 10x and scale bar of 100 μm. Negative and positive controls are represented by TCPS and cell lysis, respectively.

The fluorescent micrographs showed the presence of live cells (green) on the three different types of fibers. Furthermore, the morphology of the cells grown on the fibers was similar to the morphology of cells grown on TCPS. PHA fibers and PHA-NCO-sP(EO-stat-PO) fibers showed a slightly lower cell density as compared to the remaining types of fibers (Figure 8).

### Prevention of Bacterial Cell Adhesion on Activated Fibers

The anti-adhesive properties of the materials against bacteria was evaluated by incubating the different fibers with *S. epidermidis* (ATCC^®^ 35984^TM^) for a period of 24 h. The bacteria that adhered to the surface of the fibers was removed by sonication and quantified by plating on blood agar plates. The ability of the functionalized fibers to prevent bacterial adhesion was expressed as a percentage by comparing the number of bacteria that adhered to the surface of unmodified fibers with the number of bacteria that adhered to the surface of functionalized fibers. Additionally, after the incubation period, the biofilm biomass that was formed on the surface of the fibers was stained using the safranin method.

**FIGURE 9:**
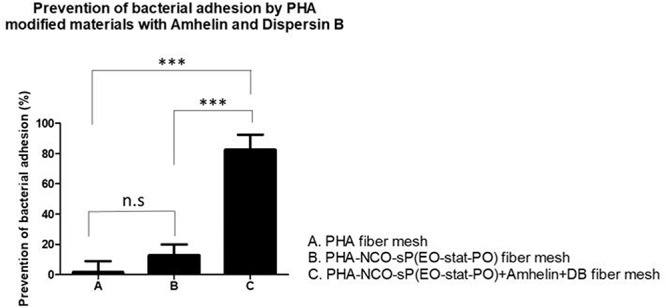
Percentage of prevention of bacterial adhesion of PHA fibers, PHA-NCO-sP(EO-stat-PO) fibers and PHA-NCO-sP(EO-stat-PO) + Amhelin + DB fibers after their incubation with *S. epidermidi*s (ATCC^®^ 35984^TM^) for a period of 24 h. The experiments were performed in two independent experiments, in triplicates (*N* = 2, *n* = 3). All columns were compared with each other. ^∗∗∗^*p* < 0.0001 and n.s indicates no statistical difference.

The results showed that PHA-NCO-sP(EO-stat-PO) + Amhelin + DB fibers were able to prevent the bacterial adhesion by 88% when compared to unmodified PHA fibers. The fibers containing NCO-sP(EO-stat-PO) only showed similar bacterial adhesion to that of the PHA fibers (Figure 9).

**FIGURE 10:**
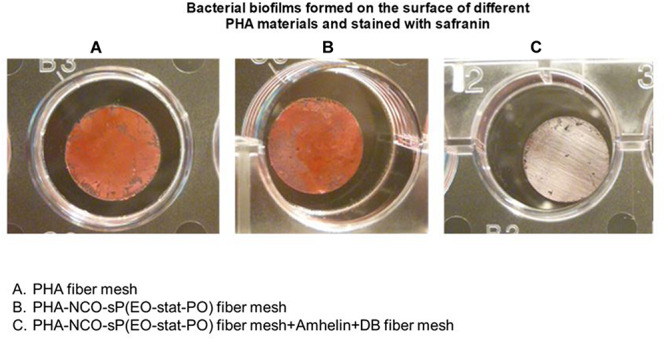
Safranin staining of bacterial biofilms formed on top of PHA fibers **(A)**, PHA-NCO-sP(EO-stat-PO) fibers **(B)** and PHA-NCO-sP(EO-stat-PO) + Amhelin + DB fibers **(C)** after a 24-hour bacterial incubation.

The staining method showed that PHA fibers and PHA-NCO-sP(EO-stat-PO) fibers were completely covered by a biofilm biomass layer while the fibers functionalized with Amhelin + DB showed the absence of *S. epidermidis* (ATCC^®^ 35984^TM^) biofilms on their surface (Figure 10).

## Discussion

Despite the success of many regenerative medicine strategies, such as tissue engineering, the problem of contamination of implantable biomaterials by bacteria persists. Bacteria can rapidly recognize a foreign material and start its adhesion process. As the implants get more and more colonized by bacteria, they start forming a protective layer which becomes much harder to treat than common planktonic bacteria. Thus, there is a strong need to mitigate bacterial adhesion on implants with features such as surface activated properties that are unfavorable for bacterial adhesion but favorable for eukaryotic cell adhesion. The development of non-cytotoxic and anti-adhesive fiber meshes was achieved by surface modifying PHA fibers with bacterial membrane targeting Amhelin and the biofilm disrupting enzyme DB.

The antibacterial screening of Amhelin showed the bacteriostatic and bactericidal nature of this AMP. According to the European Committee on Antimicrobial Susceptibility Testing (EUCAST) MIC Breakpoints, the most commonly used antibiotics such as vancomycin, gentamycin and rifampicin have a MIC of 4 mg/mL, 1 mg/mL and 60 μg/mL, respectively, against some of the most relevant pathogenic bacteria (The European Committee on Antimicrobial Susceptibility Testing, 2019). Herein, the obtained MIC and MBC values showed concentrations ranging from 6 to 12 μg/mL, suggesting an extremely good efficacy at low concentrations. Also, in previous experiments performed in our lab we observed inhibitory growth effects against a methicillin-resistant *S. epidermidis* strain, showing a potential effect against antibiotic-resistant strains.

Besides the indication of the bacteriostatic and bactericidal nature of the peptide, promising results were obtained regarding the anti-biofilm action of Amhelin as in this work, we showed for the first time that Amhelin had the ability to inhibit biofilm formation at just 3 μg/mL. The antibacterial screening showed that with concentrations equivalent to the MIC, biofilm formation was inhibited between 80 to 90% compared to the biofilm growth control.

Although the mechanism of action by which AMPs act against biofilms is not entirely understood, literature suggests that they interfere with cell communication systems such as quorum sensing, bind to polysaccharides present in the biofilm matrix, downregulate genes responsible for biofilm formation or disrupt and depolarize the membrane potential of biofilm embedded cells (Yasir et al., 2018). Since Amhelin predominantly functions by creating pores in bacterial phospholipid bilayers, it is likely that the anti-biofilm action of the peptide takes place at a membrane level.

The initial evidence that DB could promote biofilm dispersal came from studies which showed that the DB mutant (mutant strain not producing the enzyme) lacked the biofilm dispersal ability since the cells resulting from the mutant produced biofilm colonies that were not released to the medium as opposed to the wild-type strain (Kaplan et al., 2003). Other studies provided further proof that DB was indeed causing biofilm dispersal as the purified enzyme was shown to disperse cells from pre-formed biofilm colonies of bacteria. The MALDI-TOF mass spectra of the extracellular polysaccharide, after being digested with DB showed a high degree of depolymerization (Itoh et al., 2005; Izano et al., 2007). In addition, it was shown that DB could act against Staphylococci strains including *S. epidermidis* and *Staphylococcus aureus* (Kaplan et al., 2004; Izano et al., 2008). The present study confirmed the anti-biofilm activity of DB against *S. epidermidis* (ATCC^®^ 35984^TM^).

The cytocompatibility evaluation of the different agents demonstrated a higher selectivity for bacteria over mammalian cells, as the estimated MICs and MBCs were considerably lower than the CC_50_ calculated for L929 murine fibroblasts. The fact of having such a safe therapeutic window in which the agents could be used was extremely convenient if the aim was to prevent bacterial adherence to the maximum without interfering with the adherence of eukaryotic tissue cells.

Bead formation is a common problem in the electrospinning process and can affect the quality of the fibers. On the other hand, non-aligned fibers can affect the process of eukaryotic tissue cell adhesion due to differences in morphology as aligned fiber structures resemble the ECM pattern (Bourget et al., 2013). Beadless and slightly aligned fibers were obtained using the appropriate spinning conditions and parameters resulting in fibers with a suitable morphology for cell attachment, spreading and proliferation.

The surface properties of the materials such as wettability are known to play a critical role in the process of eukaryotic tissue cell adhesion, proliferation and differentiation as well as bacterial adhesion, therefore the wettability of the materials produced in this study were evaluated and its effect considered in the process of eukaryotic tissue cell adhesion and bacterial adhesion. In this study, it was shown that when PHA was processed to form electrospun fiber meshes, the WCA increased drastically to values of 120° and further increased when processed to form electrospun fiber meshes along with NCO-sP(EO-stat-PO), to a WCA of 132°, granting the materials almost superhydrophobic properties. It is known that roughness has a great influence on the water contact angle of a material, and that hydrophobic materials, when fabricated into highly rough and porous substrates, such as electrospun fiber meshes, become even more hydrophobic (Wilson et al., 2005; Yuan and Lee, 2013; Law, 2014). The fact that similar contact angles for fiber meshes with and without NCO-sP(EO-stat-PO) were obtained indicates that there was not a strong tendency for surface segregation, possibly due to a good miscibility of the additive with the polymer. On the other hand, and as expected when covalently coupling the active agents to the fibers, a much lower WCA was obtained. This evidenced the introduction of hydrophilic groups on the surface of the fibers, derived from Amhelin and DB, causing the fibers to have a significantly lower contact angle. Additionally, the results suggested that these molecules contained enough polar groups to interact with the charges during the spinning process and to promote surface segregation. Additionally, when PHA fibers with and without NCO-sP(EO-stat-PO) were incubated in a humidified atmosphere for 4 h, the water contact angle remained the same for the PHA fibers, while it decreased significantly in the case of the PHA-NCO-sP(EO-stat-PO) fibers. Isocyanate groups, NCO, are highly moisture-sensitive (Rolph et al., 2016). The samples containing NCO-sP(EO-stat-PO) when incubated in a deionized water-saturated atmosphere could have resulted in the deactivation of the NCO groups causing changes in its wettability properties caused primarily by water uptake in the polymer matrix. Further experiments assessing the stability of PHA-NCO-sP(EO-stat-PO) + Amhelin + DB fibers should also be considered to evaluate the long-term effect of the materials.

Quantitative cytocompatibility assays revealed cell viability values higher than 70% for all the electrospun fibers prepared in this study when compared to the TCPS control, showing in general, a good cytocompatibility toward L929 murine fibroblasts. Relatively hydrophilic surfaces are generally preferred by eukaryotic tissue cells as they tend to promote the attraction of proteins from the media, such as adhesion of growth factors, onto their surfaces, hence facilitating the process of cell adhesion and growth (Lampin et al., 1997; Wilson et al., 2005). For this reason, when observing the qualitative fluorescent micrographs it was seen that, the material with the lowest contact angle, that is PHA-NCOsP(EOstat PO) + Amhelin + DB fibers, 62°, not only exhibited a higher cell density, but also showed that the cells were able to attach and elongate better along the fibers, indicating that the relatively more hydrophilic properties of the fibers may have promoted the process of cell adhesion.

The colonization on bioactive fibers by *S. epidermidis* (ATCC^®^ 35984^TM^) was significantly lower than that on the unmodified PHA fibers and PHA-NCO-sP(EO-statPO) fibers, serving as an indicator that the covalent coupling of the agents had successfully conferred anti-adhesive properties to the electrospun meshes. More specifically, it prevented bacterial adhesion by more than 80% as compared to the unmodified PHA fibers and PHA-NCO-sP(EO-statPO) fibers. The safranin staining also gave an indication as to whether the bacteria that adhered to the fibers was capable of forming biofilms in a 24-hour bacterial incubation period and the results showed the absence of biofilm biomass on the bioactivated fibers, suggesting that the materials could prevent biofilm formation because as a rule of thumb, if bacteria are unable to adhere to a surface, they are unable to colonize it and form a biofilm.

As mentioned before, PNAG is responsible for the process of bacterial adhesion and although the biofilm matrix and chemical composition of biofilms can vary among bacterial strains, PNAG has been identified as the main exopolysaccharide present in Staphylococcal biofilms and other bacterial strains including *E. coli* (Wang et al., 2004; Arciola et al., 2015). Given the results obtained and given that this polysaccharide is present in the composition of biofilms of major pathogens reiterates the importance of exploring DB to tackle biofilm formation of other relevant pathogens.

Another important aspect of this study is that although still in preliminary stages, it complements the portfolio of medical materials with antibacterial properties. The chemical modification of natural and synthetic polymers with either AMPs or DB has progressively become a strategy to enhance the performance of implantable materials. For instance, Piras et al. (2015) performed the encapsulation of frog-skin derived AMP, temporin B (TB) into chitosan nanoparticles (CS-NPs) in which antibacterial action was seen against various strains of *S. epidermidis*. In a study conducted by Pavlukhina et al. (2012) the surface of poly(allylamine hydrochloride) (PAH) was modified with DB showing anti-biofilm properties against *S. epidermidis.* And, in a study conducted by Marcano et al. (2017) PHA asymmetric membranes containing DB were developed for wound healing applications showing activity against *S. epidermidis* biofilms. DB has also been combined with other agents such as antibiotic cefamandole nafate and triclosan that were physically adsorbed to the surface of polyurethanes resulting in active materials against Staphylococcal biofilms (Donelli et al., 2007; Darouiche et al., 2009). Despite encouraging results, the medical community still strives to develop alternative strategies to prevent biofilm formation on implantable materials and our study adds a newly developed 3D polymeric structure with unprecedented anti-adhesive and antibiofilm properties.

Plus, these materials could be used in a range of medical applications in the form of wound dressings, patches, tissue engineering scaffolds or medical device coatings. For instance, osteomyelitis is a condition where bones get infected and it is one of the most challenging chronic infections found in patients with open fractures and in patients requiring orthopedic procedures. Usually, its treatment includes high doses of antibiotics for long periods of time, surgery and debridement. As an example, when bacteria infect a bone, the blood flow of the infected area decreases and necrotic bone or dead bone is formed, called the sequestrum. In cases where a sequestrum is formed, surgical debridement is carried out to remove the sequestrum but often other tissues are affected and can get damaged such as the periosteum. The periosteum is a membrane covering the bone structure which provides nutrients and also serves as a reservoir of mesenchymal stem cells. If the periosteum is not repaired after surgery, there is a delay in the healing process accompanied with lack of nutrients and a much slower and compromised regeneration process. Therefore, one of the applications of such fiber meshes could be as a periosteum patch which would aid in the regenerative process and at the same time prevents further bacterial contaminations. Additionally, it sometimes happens that after the removal of the sequestrum, a large bone defect is present which is usually compensated by the introduction of intermedullary titanium nails. However, the prevention of infections is compromised as because of the absence of vascularization, the antibiotics cannot reach this area (Berebichez-Fridman et al., 2017). Therefore, one of the applications of the materials developed here could be as a coating of such nails.

To achieve this, further in-depth studies are required. For instance, further characterization steps are needed and are here suggested as future work. Chemical analysis such as X-ray photoelectron spectroscopy, atomic force microscopy or Attenuated total reflectance-Fourier transform infrared spectrometry should be employed to detect the presence of the agents on the fibers as well as its spatial topographical distribution and to quantify the amount of agent present in the fibers. *In vitro* screening of the biodegradation profile would also be very important to evaluate the stability of the materials and finally an *in vitro* release profile of the antimicrobials should also be investigated. Nonetheless, our proof of principle study showed a straightforward, one-step procedure to engineer bioactive fiber meshes based on PHAs and demonstrated that the idea of combining different novel and innovative antibacterial agents with different mechanisms of action against bacteria could be a promising approach toward the development of effective and efficient anti-biofilm therapies.

## Data Availability Statement

All datasets generated for this study are included in the article/supplementary material.

## Author Contributions

SP: original draft preparation. SP, JS, and IR: design of the study, investigation. LM: support and revision of experiments. RV: critical proofreading. HL, MR, and JG: supply of antimicrobial agents. MR, JG, JS, and IR: revision of the manuscript and provision of intellectual contributions to the manuscript. JS, IR: funding acquisition.

## Conflict of Interest

The authors declare that the research was conducted in the absence of any commercial or financial relationships that could be construed as a potential conflict of interest.
